# Bis[μ-3-(1*H*-benzimidazol-2-yl)benzoato]dicopper(I)

**DOI:** 10.1107/S1600536810046040

**Published:** 2010-11-17

**Authors:** Ke-Wei Lei, Dong-Guo Xia, Jie Li, Zheng-Yu Su

**Affiliations:** a State Key Laboratory Base of Novel Functional Materials and Preparation Science, Institute of Solid Materials Chemistry, Faculty of Materials Science and Chemical Engineering, Ningbo University, Ningbo 315211, People’s Republic of China

## Abstract

The dimeric title complex, [Cu_2_(C_14_H_9_N_2_O_2_)_2_], resides on a center of symmetry. In the crystal, the mol­ecules are packed *via* π–π stacking inter­actions alternating between imidazole and benzene rings [mean inter­planar distances = 3.754 (3) and 3.624 (3) Å]. An inter­molecular N—H⋯O hydrogen bond links the dimers together. The two-coordinate Cu^I^ atom displays an O—Cu—N bond angle of 176.3 (2)°. The Cu⋯Cu distance within the dimer is 5.100 (2) Å.

## Related literature

For background to complexes of benzimidazole with copper(I), see: Ruettimann *et al.* (1992 ▶).
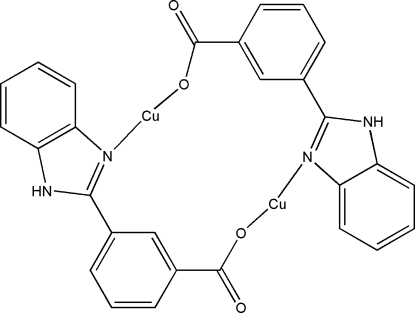


## Experimental

### 

#### Crystal data

[Cu_2_(C_14_H_9_N_2_O_2_)_2_]
*M* *_r_* = 601.54
Monoclinic, 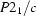

*a* = 4.875 (2) Å
*b* = 13.417 (7) Å
*c* = 18.130 (9) Å
β = 103.643 (12)°
*V* = 1152.4 (10) Å^3^
*Z* = 2
Mo *K*α radiation
μ = 1.89 mm^−1^
*T* = 296 K
0.52 × 0.44 × 0.40 mm

#### Data collection

Bruker SMART APEXII diffractometer
Absorption correction: multi-scan (*SADABS*; Sheldrick, 2000 ▶) *T*_min_ = 0.387, *T*_max_ = 0.473
7794 measured reflections
2013 independent reflections
1083 reflections with *I* > 2σ(*I*)
*R*_int_ = 0.107

#### Refinement

*R*[*F* ^2^ > 2σ(*F* ^2^)] = 0.056
*wR*(*F* ^2^) = 0.148
*S* = 1.01
2013 reflections
172 parameters
H-atom parameters constrained
Δρ_max_ = 0.69 e Å^−3^
Δρ_min_ = −0.62 e Å^−3^

### 

Data collection: *APEX2* (Bruker, 2007 ▶); cell refinement: *SAINT* (Bruker, 2007 ▶); data reduction: *SAINT*; program(s) used to solve structure: *SHELXS97* (Sheldrick, 2008 ▶); program(s) used to refine structure: *SHELXL97* (Sheldrick, 2008 ▶); molecular graphics: *SHELXTL* (Sheldrick, 2008 ▶); software used to prepare material for publication: *SHELXL97*.

## Supplementary Material

Crystal structure: contains datablocks global, I. DOI: 10.1107/S1600536810046040/om2365sup1.cif

Structure factors: contains datablocks I. DOI: 10.1107/S1600536810046040/om2365Isup2.hkl

Additional supplementary materials:  crystallographic information; 3D view; checkCIF report

## Figures and Tables

**Table:** 

Cu1—O2	1.823 (5)
Cu1—N2^i^	

**Table:** 

O2—Cu1—N2^i^	176.3 (2)

Symmetry code: (i) 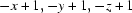.

**Table 2:** Hydrogen-bond geometry (Å, °)

*D*—H⋯*A*	*D*—H	H⋯*A*	*D*⋯*A*	*D*—H⋯*A*
N1—H1⋯O1^ii^	0.86	1.95	2.783 (7)	164

Symmetry code: (ii) 
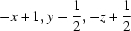
.

**Fig. 1.**
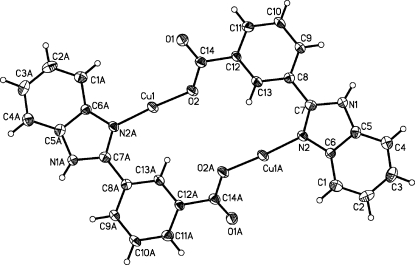
The structure of the title compound, showing 30% probability displacement ellipsoids and the atom-numbering scheme.

**Fig. 2.**
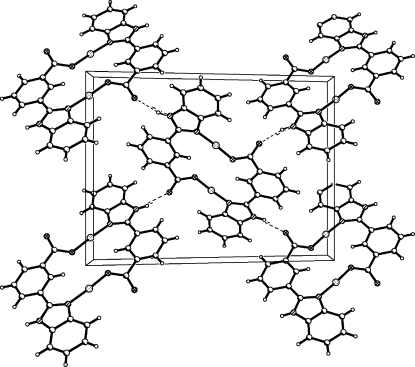
Packing diagram of the title compound.

## supplementary crystallographic information

### Comment

The title complex, [Cu(C_14_H_9_N_2_O_2_)]_2_, is a dimer and resides on a center of symmetry. The molecular structure is illustrated in Fig. 1. Due to the low coordination number of two for copper(I), the Cu(I)–N and Cu(I)–O bond lengths are somewhat shorter than typical values (Ruettimann *et al.*, 1992).

The dihedral angle between the two planes of C1–C6 and C8–C13 benzene rings is 4.37 (3)°. In the title molecule there is an intermolecular hydrogen bond between N1 and O1 of an adjacent dimer (Table 2 and Fig. 2). The mean interplanar distance of 3.754 (3)Å and 3.624 (3)Å alternately between imidazole and benzene rings suggests that the ligands are engaged in π-π stacking interactions with an offset face-to-face style.

### Experimental

Under microwave irradiation, 3-(1*H*-benzo[*d*]imidazol-2-yl) benzoic acid was synthesized by condensation of benzene-1,2-diamine and isophthalic acid(1:1) in polyphosphoric acid. The reaction time was 12 min. The yield was 74%. Then recrystallization from methanol gave pure chemical compound. A mixture of 3-(1*H*-benzo[*d*]imidazol-2-yl)benzoic acid (0.0476 g,0.2 mmol),Cu(NO_3_)_2_.3H_2_O (0.121 g, 0.5 mmol) and and water (l2 ml) was placed in a Teflon-lined stainless steel vessel (25 ml) and heated at 170 °C for 72 h, and then cooled to room temperature at a rate of 0.1 °C/min. The resulting brown single crystals were isolated by washing with DMF/methanol, and dried *in vacuo*. It can be seen from the crystal color that the Cu^2+^ ion was reduced from +2 to +1 oxidation state. The yield was about 37 mg (62 %). Calcd.for C_28_H_18_Cu_2_N_4_O_4_: C, 55.86; H, 2.99; N, 9.31; Found: C, 55.92;H, 2.93;N, 9.24%.

### Refinement

All H atoms were placed in geometrically idealized positions and treated as riding on their parent atoms, with C—H = 0.93–0.96 Å, and with *U*_iso_(H) = 1.2*U*_eq_(C) or 1.5*U*_eq_(C) for methyl H atoms.

### Crystal data

**Table:**

[Cu_2_(C_14_H_9_N_2_O_2_)_2_]	*F*(000) = 608
*M**_r_* = 601.54	*D*_x_ = 1.734 Mg m^−^^3^
Monoclinic, *P*2_1_/*c*	Mo *K*α radiation, λ = 0.71073 Å
Hall symbol: -P 2ybc	Cell parameters from 1146 reflections
*a* = 4.875 (2) Å	θ = 2.3–21.7°
*b* = 13.417 (7) Å	µ = 1.89 mm^−^^1^
*c* = 18.130 (9) Å	*T* = 296 K
β = 103.643 (12)°	Block, brown
*V* = 1152.4 (10) Å^3^	0.52 × 0.44 × 0.40 mm
*Z* = 2	

### Data collection

**Table:**

Bruker SMART APEXII diffractometer	2013 independent reflections
Radiation source: fine-focus sealed tube	1083 reflections with *I* > 2σ(*I*)
graphite	*R*_int_ = 0.107
φ and ω scans	θ_max_ = 25.0°, θ_min_ = 2.3°
Absorption correction: multi-scan (*SADABS*; Sheldrick, 2000)	*h* = −5→4
*T*_min_ = 0.387, *T*_max_ = 0.473	*k* = −15→15
7794 measured reflections	*l* = −21→21

### Refinement

**Table:**

Refinement on *F*^2^	Primary atom site location: structure-invariant direct methods
Least-squares matrix: full	Secondary atom site location: difference Fourier map
*R*[*F*^2^ > 2σ(*F*^2^)] = 0.056	Hydrogen site location: inferred from neighbouring sites
*wR*(*F*^2^) = 0.148	H-atom parameters constrained
*S* = 1.01	*w* = 1/[σ^2^(*F*_o_^2^) + (0.0617*P*)^2^ + 0.5546*P*] where *P* = (*F*_o_^2^ + 2*F*_c_^2^)/3
2013 reflections	(Δ/σ)_max_ < 0.001
172 parameters	Δρ_max_ = 0.69 e Å^−^^3^
0 restraints	Δρ_min_ = −0.62 e Å^−^^3^

### Special details

**Table:**

Geometry. All e.s.d.'s (except the e.s.d. in the dihedral angle between two l.s. planes) are estimated using the full covariance matrix. The cell e.s.d.'s are taken into account individually in the estimation of e.s.d.'s in distances, angles and torsion angles; correlations between e.s.d.'s in cell parameters are only used when they are defined by crystal symmetry. An approximate (isotropic) treatment of cell e.s.d.'s is used for estimating e.s.d.'s involving l.s. planes.
Refinement. Refinement of *F*^2^ against ALL reflections. The weighted *R*-factor *wR* and goodness of fit *S* are based on *F*^2^, conventional *R*-factors *R* are based on *F*, with *F* set to zero for negative *F*^2^. The threshold expression of *F*^2^ > σ(*F*^2^) is used only for calculating *R*-factors(gt) *etc*. and is not relevant to the choice of reflections for refinement. *R*-factors based on *F*^2^ are statistically about twice as large as those based on *F*, and *R*- factors based on ALL data will be even larger.

### Fractional atomic coordinates and isotropic or equivalent isotropic displacement parameters (Å^2^)

**Table:**

	*x*	*y*	*z*	*U*_iso_*/*U*_eq_	
Cu1	0.09380 (19)	0.62278 (7)	0.49047 (4)	0.0455 (3)	
O1	0.0431 (11)	0.6329 (4)	0.3136 (3)	0.0546 (13)	
O2	0.2212 (11)	0.5488 (4)	0.4211 (2)	0.0528 (14)	
N1	1.0395 (10)	0.2257 (4)	0.3273 (3)	0.0336 (13)	
H1	0.9989	0.2078	0.2804	0.040*	
N2	1.0206 (11)	0.3061 (4)	0.4335 (3)	0.0340 (13)	
C1	1.4326 (14)	0.2163 (5)	0.5182 (4)	0.0433 (18)	
H1A	1.4275	0.2497	0.5628	0.052*	
C2	1.6298 (14)	0.1429 (6)	0.5170 (4)	0.051 (2)	
H2	1.7583	0.1263	0.5620	0.061*	
C3	1.6418 (15)	0.0926 (5)	0.4499 (5)	0.053 (2)	
H3	1.7786	0.0438	0.4516	0.064*	
C4	1.4583 (14)	0.1134 (5)	0.3825 (4)	0.0433 (18)	
H4	1.4664	0.0801	0.3381	0.052*	
C5	1.2557 (13)	0.1879 (5)	0.3836 (4)	0.0363 (16)	
C6	1.2412 (13)	0.2388 (5)	0.4501 (4)	0.0343 (16)	
C7	0.9009 (13)	0.2964 (5)	0.3587 (3)	0.0339 (16)	
C8	0.6567 (13)	0.3523 (4)	0.3147 (3)	0.0317 (16)	
C9	0.5412 (14)	0.3314 (5)	0.2385 (4)	0.0407 (17)	
H9	0.6192	0.2812	0.2145	0.049*	
C10	0.3124 (14)	0.3844 (5)	0.1981 (4)	0.0472 (19)	
H10	0.2345	0.3689	0.1475	0.057*	
C11	0.1979 (14)	0.4609 (5)	0.2330 (4)	0.0419 (18)	
H11	0.0468	0.4978	0.2053	0.050*	
C12	0.3074 (13)	0.4824 (4)	0.3086 (3)	0.0311 (15)	
C13	0.5339 (13)	0.4285 (5)	0.3486 (4)	0.0347 (16)	
H13	0.6073	0.4432	0.3996	0.042*	
C14	0.1782 (14)	0.5627 (5)	0.3490 (4)	0.0378 (17)	

### Atomic displacement parameters (Å^2^)

**Table:**

	*U*^11^	*U*^22^	*U*^33^	*U*^12^	*U*^13^	*U*^23^
Cu1	0.0639 (6)	0.0446 (6)	0.0288 (5)	0.0096 (5)	0.0128 (4)	−0.0037 (4)
O1	0.075 (4)	0.049 (3)	0.042 (3)	0.021 (3)	0.018 (3)	0.008 (2)
O2	0.083 (4)	0.050 (3)	0.026 (3)	0.022 (3)	0.014 (2)	0.002 (2)
N1	0.035 (3)	0.036 (3)	0.031 (3)	0.002 (3)	0.009 (3)	−0.002 (3)
N2	0.045 (3)	0.032 (3)	0.027 (3)	0.000 (3)	0.012 (3)	0.000 (2)
C1	0.050 (4)	0.042 (4)	0.039 (4)	−0.008 (4)	0.012 (3)	0.005 (3)
C2	0.039 (4)	0.053 (5)	0.054 (5)	−0.005 (4)	−0.002 (4)	0.017 (4)
C3	0.046 (5)	0.039 (5)	0.077 (6)	0.009 (4)	0.019 (4)	0.008 (4)
C4	0.048 (4)	0.038 (4)	0.047 (4)	−0.002 (4)	0.018 (3)	−0.001 (4)
C5	0.034 (4)	0.035 (4)	0.043 (4)	−0.002 (3)	0.018 (3)	0.004 (3)
C6	0.032 (4)	0.040 (4)	0.031 (4)	0.003 (3)	0.009 (3)	0.003 (3)
C7	0.039 (4)	0.033 (4)	0.031 (4)	−0.007 (3)	0.012 (3)	0.005 (3)
C8	0.035 (4)	0.029 (4)	0.032 (4)	−0.003 (3)	0.012 (3)	0.004 (3)
C9	0.046 (4)	0.046 (4)	0.031 (4)	0.006 (4)	0.009 (3)	−0.009 (3)
C10	0.054 (5)	0.060 (5)	0.026 (4)	0.006 (4)	0.009 (3)	−0.013 (4)
C11	0.045 (4)	0.048 (5)	0.032 (4)	0.002 (4)	0.009 (3)	0.003 (3)
C12	0.039 (4)	0.031 (4)	0.026 (3)	0.001 (3)	0.013 (3)	−0.001 (3)
C13	0.044 (4)	0.031 (4)	0.029 (4)	−0.002 (3)	0.010 (3)	−0.004 (3)
C14	0.048 (4)	0.036 (4)	0.031 (4)	−0.001 (4)	0.011 (3)	0.001 (3)

### Geometric parameters (Å, °)

**Table:**

Cu1—O2	1.823 (5)	C3—H3	0.9300
Cu1—N2^i^	1.867 (5)	C4—C5	1.408 (9)
O1—C14	1.239 (8)	C4—H4	0.9300
O2—C14	1.287 (7)	C5—C6	1.402 (9)
N1—C7	1.366 (8)	C7—C8	1.472 (8)
N1—C5	1.379 (8)	C8—C9	1.393 (9)
N1—H1	0.8600	C8—C13	1.399 (9)
N2—C7	1.349 (8)	C9—C10	1.378 (9)
N2—C6	1.382 (8)	C9—H9	0.9300
N2—Cu1^i^	1.867 (5)	C10—C11	1.391 (9)
C1—C2	1.380 (9)	C10—H10	0.9300
C1—C6	1.394 (9)	C11—C12	1.378 (8)
C1—H1A	0.9300	C11—H11	0.9300
C2—C3	1.405 (11)	C12—C13	1.375 (8)
C2—H2	0.9300	C12—C14	1.520 (9)
C3—C4	1.363 (10)	C13—H13	0.9300
			
O2—Cu1—N2^i^	176.3 (2)	C1—C6—C5	119.8 (6)
C14—O2—Cu1	128.4 (4)	N2—C7—N1	110.3 (5)
C7—N1—C5	108.3 (5)	N2—C7—C8	126.8 (6)
C7—N1—H1	125.9	N1—C7—C8	122.9 (5)
C5—N1—H1	125.9	C9—C8—C13	117.9 (6)
C7—N2—C6	106.6 (5)	C9—C8—C7	121.5 (6)
C7—N2—Cu1^i^	131.1 (5)	C13—C8—C7	120.6 (6)
C6—N2—Cu1^i^	121.7 (4)	C10—C9—C8	120.7 (6)
C2—C1—C6	117.7 (7)	C10—C9—H9	119.7
C2—C1—H1A	121.2	C8—C9—H9	119.7
C6—C1—H1A	121.2	C9—C10—C11	120.1 (6)
C1—C2—C3	121.8 (7)	C9—C10—H10	120.0
C1—C2—H2	119.1	C11—C10—H10	120.0
C3—C2—H2	119.1	C12—C11—C10	120.2 (6)
C4—C3—C2	121.7 (7)	C12—C11—H11	119.9
C4—C3—H3	119.1	C10—C11—H11	119.9
C2—C3—H3	119.1	C13—C12—C11	119.3 (6)
C3—C4—C5	116.6 (7)	C13—C12—C14	119.4 (5)
C3—C4—H4	121.7	C11—C12—C14	121.3 (6)
C5—C4—H4	121.7	C12—C13—C8	121.8 (6)
N1—C5—C6	105.8 (6)	C12—C13—H13	119.1
N1—C5—C4	131.8 (6)	C8—C13—H13	119.1
C6—C5—C4	122.4 (6)	O1—C14—O2	125.2 (6)
N2—C6—C1	131.2 (6)	O1—C14—C12	121.2 (6)
N2—C6—C5	109.0 (5)	O2—C14—C12	113.6 (6)
			
N2^i^—Cu1—O2—C14	−169 (3)	C5—N1—C7—N2	0.2 (7)
C6—C1—C2—C3	0.6 (10)	C5—N1—C7—C8	−179.8 (5)
C1—C2—C3—C4	−0.3 (11)	N2—C7—C8—C9	−176.0 (6)
C2—C3—C4—C5	−0.1 (10)	N1—C7—C8—C9	4.1 (9)
C7—N1—C5—C6	0.0 (7)	N2—C7—C8—C13	4.1 (10)
C7—N1—C5—C4	179.0 (7)	N1—C7—C8—C13	−175.8 (6)
C3—C4—C5—N1	−178.7 (7)	C13—C8—C9—C10	−0.2 (10)
C3—C4—C5—C6	0.1 (10)	C7—C8—C9—C10	180.0 (6)
C7—N2—C6—C1	−179.1 (7)	C8—C9—C10—C11	1.3 (11)
Cu1^i^—N2—C6—C1	−6.9 (10)	C9—C10—C11—C12	−1.8 (10)
C7—N2—C6—C5	0.4 (7)	C10—C11—C12—C13	1.1 (10)
Cu1^i^—N2—C6—C5	172.5 (4)	C10—C11—C12—C14	−177.3 (6)
C2—C1—C6—N2	178.8 (6)	C11—C12—C13—C8	0.0 (10)
C2—C1—C6—C5	−0.6 (10)	C14—C12—C13—C8	178.5 (6)
N1—C5—C6—N2	−0.2 (7)	C9—C8—C13—C12	−0.5 (9)
C4—C5—C6—N2	−179.3 (5)	C7—C8—C13—C12	179.4 (6)
N1—C5—C6—C1	179.3 (6)	Cu1—O2—C14—O1	1.9 (11)
C4—C5—C6—C1	0.2 (10)	Cu1—O2—C14—C12	−177.3 (4)
C6—N2—C7—N1	−0.4 (7)	C13—C12—C14—O1	156.2 (6)
Cu1^i^—N2—C7—N1	−171.5 (4)	C11—C12—C14—O1	−25.4 (10)
C6—N2—C7—C8	179.7 (6)	C13—C12—C14—O2	−24.5 (9)
Cu1^i^—N2—C7—C8	8.6 (10)	C11—C12—C14—O2	153.9 (7)

Symmetry codes: (i) −*x*+1, −*y*+1, −*z*+1.

### Hydrogen-bond geometry (Å, °)

**Table:**

*D*—H···*A*	*D*—H	H···*A*	*D*···*A*	*D*—H···*A*
N1—H1···O1^ii^	0.86	1.95	2.783 (7)	164

Symmetry codes: (ii) −*x*+1, *y*−1/2, −*z*+1/2.
